# Causal association between gut microbiota and neonatal digestive system diseases: A Mendelian randomization study

**DOI:** 10.1097/MD.0000000000044009

**Published:** 2025-08-15

**Authors:** Jian Pang, Zuojian Yang, Pingping Liu, Baoxing Pan, Guidan He, Shuihua Yang

**Affiliations:** a Department of Medical Ultrasonics, Maternal and Child Health Care of Guangxi Zhuang Autonomous Region Hospital, Nanning, Guangxi, China.

**Keywords:** causal association, genome-wide association study polymorphisms, gut microbiota, Mendelian randomization, neonatal digestive system diseases, single nucleotide polymorphisms in gut microbiota

## Abstract

To delve into the underlying causal connections that may exist between the intestinal flora and disorders of the neonatal digestive system (DSD), aiming to identify specific pathogenic bacteria associated with the development and progression of neonatal DSD. We collected summary statistics from genome-wide association studies on neonatal DSD and gut microbiome-related phenotypes from the Finngen and MiBioGen consortia. We conducted a bidirectional, two-sample Mendelian randomization (MR) analysis to detect causal relationships between the gut microbiome and neonatal DSD. Data on 211 gut microbiome species were obtained from samples of various ethnicities (16,380,466 samples). DSD data were sourced from 143 cases and 218,649 control samples of European ancestry. Single nucleotide polymorphisms were chosen as instrumental variables in accordance with precise selection criteria. We primarily employed inverse variance weighted meta-analysis, supplemented by weighted median, MR-Egger, weighted mode, and simple mode analyses. Results were assessed using odds ratios (OR) and 95% confidence intervals (CI). Sensitivity analyses, including heterogeneity tests, horizontal pleiotropy tests, and Steiger directionality tests, were performed to rigorously examine and validate the stability and reliability of our findings. The inverse variance weighted methodology yields strong indications of a direct causality between 6 identified microbial species and neonatal DSD. Genus Lachnospiraceae UCG010, genus *Peptococcus*, and genus Tyzzerella3 Correlated with a heightened likelihood of neonatal DSD, with genus Lachnospiraceae UCG010 showing the highest risk (OR = 4.49, *P* = .034, 95% CI [1.13–17.89]). Conversely, Bacilli, genus *Allisonella*, and phylum Proteobacteria were associated with a reduced risk of neonatal DSD, with phylum Proteobacteria showing the lowest risk (OR = 0.23, *P* = .03, 95% CI [0.06–0.88]). Q-tests indicated no heterogeneity in these results, and MR-Egger tests showed no significant horizontal pleiotropy. Leave-one-out sensitivity analyses did not identify any Single nucleotide polymorphisms with a substantial impact on the overall results. Our study elucidates a clear causal relationship between the gut microbiome and neonatal DSD, identifying 6 specific microbial taxa with potential pathogenic roles. These results offer novel perspectives on potential therapeutic interventions and the underlying processes of neonatal DSD.

## 1. Introduction

A balanced gut microbiome in early neonatal life is crucial for physiological functions and immune system maturation.^[1–3]^ Recent evidence highlights the gut microbiome role in providing resistance to colonization by pathogens or opportunistic gut-derived pathogens. Dysbiosis in the gut microbiota can predispose neonates to digestive system diseases (DSD).^[4]^ It has been demonstrated through studies that the genetic makeup influences the gut microbiome composition, with some bacterial species exhibiting inheritable traits.^[5,6]^ In the neonatal gastrointestinal tract, the microbiome is a key player in a variety of diseases^[7]^ and could be directly related to the initiation and advancement of gastrointestinal inflammation and cancerous conditions.^[8,9]^ Increasing evidence suggests that specific gut bacteria communication is associated with the occurrence of gastrointestinal cancers.^[10]^ Variations in the gut microbiota have been correlated with chronic digestive disorders, including inflammatory bowel conditions and colorectal malignancies.^[11–13]^ However, The nuanced relationship between the intestinal microbiota and neonatal DSD is still not comprehensively elucidated. Therefore, further research is essential for delving into the potential causal links between the gut microbiome and neonatal DSD.

The preponderance of studies examining the gut microbiome utilizes observational designs, making them susceptible to the influence of confounders and the challenge of disentangling causal direction.^[14]^ The capability of observational studies to determine causality is often limited due to the potential for confounding from variables that have been measured or, more problematically, those that have not been measured, which can bias the observed relationships.^[15]^ Furthermore, the interplay between the gut microbiome and the host’s health status complicates the analysis of findings from observational studies.^[16]^ These investigations contribute important perspectives on the relationships linking the gut microbiome to diverse pathological conditions, although these studies offer valuable insights, they do not conclusively establish causality. Randomized controlled trials are crucial for mitigating the impact of confounding variables and delivering compelling evidence of the interplay between the gut microbiome and disease states.^[17,18]^ Nevertheless, conducting randomized controlled trials demands a significant number of subjects and the application of intricate data analysis procedures. The financial burden, time restrictions, and ethical concerns significantly challenge the field of microbiome research, thereby complicating the establishment of causal relationships.^[19,20]^ Thus, opting for appropriate research methods to uncover the causal link between the gut microbiome and gastrointestinal disorders is exceptionally significant.

Mendelian randomization (MR) employs genetic differences to create instrumental variables (IVs) for assessing causal associations between exposures and health outcomes.^[21]^ Since the distribution of genotypes from parents to offspring is random, MR can be seen as an alternative to randomized controlled trials, with the advantage that the genetic variation-exposure association is not affected by common confounders, ensuring a reasonable causal sequence.^[22,23]^ To date, no published research has yet employed MR to explore the causal connections between gut microbiota and neonatal DSD.

The objective of this research is to execute a two-sample MR study to determine the causality between the gut microbiome and neonatal DSD, and to identify gut microbes associated with this disease.

## 2. Materials and methods

### 2.1. Data sources

The genetic variations of the gut microbiome in our research were ascertained through a meta-analysis of genome-wide association studies on gut microbiome composition, conducted by the MiBioGen consortium (https://mibiogen.gcc.rug.nl/menu/main/home/).^[24]^ This analysis included data on 211 gut microbiota species, comprising 15 unnamed gut microbiota and 196 specific bacterial taxa, which include 9 phyla, 16 classes, 20 orders, 32 families, and 119 genera, covering a total of 16,380,466 relevant single nucleotide polymorphisms (SNPs).

Data on neonatal DSD were sourced from the FinnGen consortium (R9 release) genome-wide association studies results, which included 143 cases and 218,649 controls (https://www.finngen.fi/en/access_results).^[25]^ Given that this research employed summary data that are publicly available, additional ethical permissions or consents from participants were not required.

### 2.2. Selection of IVs

The selection of genetic variants as IVs was based on their association with the exposure factor, which is the gut microbiome. In order to detect SNPs linked to the exposure and substantiate the reliability and exactitude of the conclusions regarding the causal relationship between the gut microbiome and the risk of neonatal DSD, the following methodologies were applied^[26,27]^:

Significance threshold: SNPs were identified based on a stringent genome-wide significance level of (*P* < 5 × 10^−8^) pertinent to gut microbiome studies.

Linkage disequilibrium (LD): To avoid bias due to residual LD among selected SNPs, a distance threshold of 10,000 kb and an LD *r*^2^ < 0.001 were applied.

Lowest *P*-values: SNPs were chosen based on their association with the gut microbiome, with preference given to those with the lowest *P*-values.

In this study, the SNPs with notable associations to the gut microbiome were selected to serve as IVs, with neonatal DSD designated as the outcome measure. To appropriately select IVs for the two-sample MR study, 2 key assumptions were made:

Relevance: IVs must have a significant association with the gut microbiome.

Exclusion restriction: IVs must not directly affect the outcome but can only influence it indirectly through their association with the gut microbiome.

**Figure 1.**
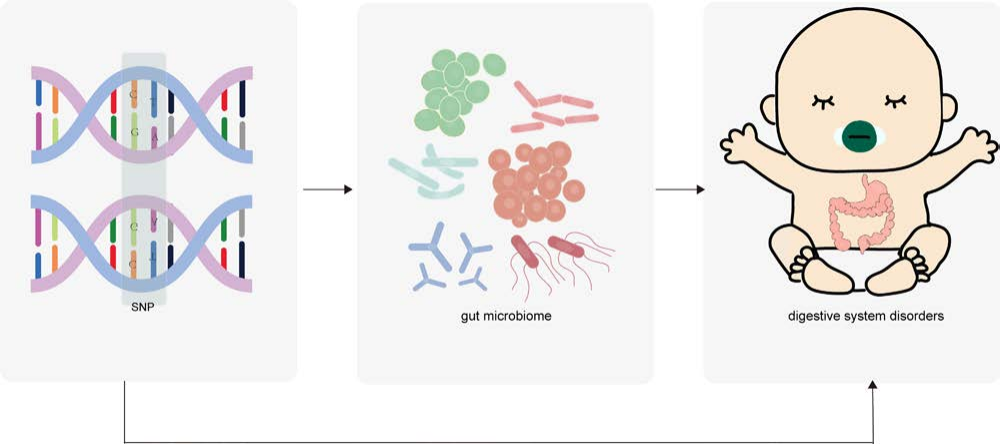
Mendelian randomization analysis assumptions.

These assumptions are illustrated in Figure 1.

### 2.3. MR analysis

The statistical analysis was executed with the aid of the TwoSampleMR package in the R environment, version 4.2.1 (https://github.com/MRCIEU/TwoSampleMR). The relationship between the gut microbiome and neonatal DSD was analyzed using the inverse variance weighted (IVW) method. The IVW approach involved regressing the SNP-gut microbiome associations against the SNP-neonatal DSD associations to obtain the IVW mean of the SNP ratio estimates.^[28]^ Complementary analytical techniques, including the weighted median estimator (WME), MR-Egger, as well as simple and weighted mode assessments, were also engaged. Results were expressed as odds ratios (OR) with 95% confidence intervals (CI), and a *P*-value of <.05 was considered statistically significant.^[29]^

### 2.4. Sensitivity analysis

Sensitivity analyses included tests for heterogeneity and directional horizontal pleiotropy. Horizontal pleiotropy was assessed using the MR-Egger regression test.^[30]^ The lack of significance in the MR-Egger intercept (*P* > .05) implies that horizontal pleiotropy is not present. Cochran Q test was employed to evaluate the variability among the SNPs. A positive finding in Cochran Q test (*P* ≤ .05) indicates considerable heterogeneity among the study’s findings. The Steiger directionality test was used to assess the robustness of the study findings by reversing the inference direction.^[31]^ Furthermore, a leave-one-out approach was utilized to assess whether any individual SNP was primarily responsible for the significant results. The correlation of the gut microbiome with the risk for neonatal DSD was expressed in terms of OR, complemented by 95% CI. A *P*-value of ≤ 0.05 provided evidence of a possible causal relationship.^[32]^

### 2.5. Statistical analysis

Given the exploratory nature of this study, we report nominal *P*-values without multiple-testing correction to maximize detection sensitivity for hypothesis generation.

## 3. Results

**Figure 2.**
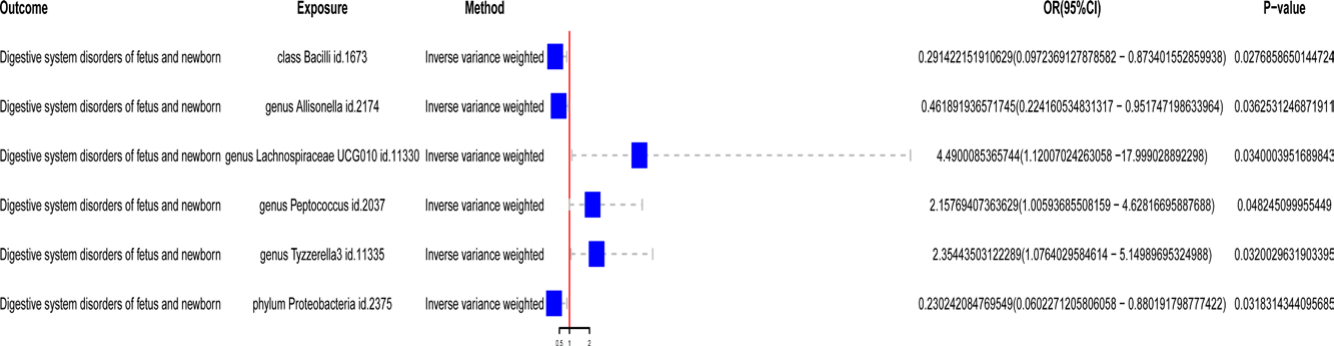
Forest plot illustrating the Mendelian randomization analysis of associations between gut microbiota and neonatal DSD. If the *P*-value is <.05, it is generally considered statistically significant. If the OR value is >1, the exposure is associated with a higher likelihood of the outcome occurring. Conversely, if the OR value is <1, the exposure is associated with a lower likelihood of the outcome, suggesting a protective effect against the outcome. OR: odds ratio.

Through MR analysis using the IVW method, we identified 6 gut microbiota taxa associated with neonatal DSD (Fig. S1, Supplemental Digital Content, https://links.lww.com/MD/P712). These include 1 class (Bacilli), 1 phylum (Proteobacteria), and 4 genera (*Allisonella*, Lachnospiraceae UCG010, *Peptococcus*, Tyzzerella3). We found that increased abundance of class Bacilli, genus *Allisonella*, and phylum Proteobacteria was associated with a reduced risk of neonatal DSD (OR = 0.29, OR = 0.46, and OR = 0.23, respectively). Conversely, increased abundance of genus Lachnospiraceae UCG010, genus *Peptococcus*, and genus Tyzzerella3 was associated with an increased risk of neonatal DSD (Table 1 and Fig. 2). Among these, genus Lachnospiraceae UCG010 showed the highest risk association with neonatal DSD (OR = 4.49).

**Table 1:** MR analysis of gut microbiome and digestive system disorders of fetus and newborn.

exposure_id	Exposure	Outcome	Method	nsnp	b	se	*P* Value	lo_ci	up_ci	or	or_lci95	or_uci95
GCST90016910	Class Bacilli	Digestive system disorders of fetus and newborn	Inverse variance weighted	18	−1.232982369	0.560011484	.027685865	−2.330604878	−0.13535986	0.291422152	0.097236913	0.873401553
GCST90016910	Class Bacilli	Digestive system disorders of fetus and newborn	MR Egger	18	−0.1625282	1.500065827	.915067257	−3.102657221	2.777600821	0.84999212	0.044929656	16.08039489
GCST90016910	Class Bacilli	Digestive system disorders of fetus and newborn	Weighted median	18	−0.994073222	0.781520943	.203382857	−2.525854272	0.537707827	0.370066255	0.07998995	1.712077982
GCST90016910	Class Bacilli	Digestive system disorders of fetus and newborn	Simple mode	18	−0.333333333	NA	.237884521	NA	NA	0.716531311	NA	NA
GCST90016910	Class Bacilli	Digestive system disorders of fetus and newborn	Weighted mode	18	−0.988652826	2.511681606	.693860228	−5.911548775	3.934243122	0.372077607	0.00270799	51.12344112
GCST90016963	Genus *Allisonella*	Digestive system disorders of fetus and newborn	Inverse variance weighted	8	−0.772424319	0.368861475	.036253125	−1.49539281	−0.049455827	0.461891937	0.224160535	0.951747199
GCST90016963	Genus *Allisonella*	Digestive system disorders of fetus and newborn	MR Egger	8	−4.224018776	2.507758453	.143091177	−9.139225344	0.691187792	0.014639692	0.00010737	1.996085059
GCST90016963	Genus *Allisonella*	Digestive system disorders of fetus and newborn	Weighted median	8	−0.333329274	0.506401046	.510389191	−1.325875323	0.659216776	0.716534219	0.265570397	1.933277551
GCST90016963	Genus *Allisonella*	Digestive system disorders of fetus and newborn	Simple mode	8	−0.25	NA	.7265625	NA	NA	0.778800783	NA	NA
GCST90016963	Genus *Allisonella*	Digestive system disorders of fetus and newborn	Weighted mode	8	−0.761562344	2.425404003	.753525583	−5.515354191	3.992229503	0.466936342	0.004024502	54.17553933
GCST90017028	Genus Lachnospiraceae UCG010	Digestive system disorders of fetus and newborn	Inverse variance weighted	10	1.501854603	0.708399593	.034000395	1.13391399974841	17.8903178060129	4.490008537	1.120070243	17.99902889
GCST90017028	Genus Lachnospiraceae UCG010	Digestive system disorders of fetus and newborn	MR Egger	10	−0.669271329	2.183816578	.767072425	−4.949551822	3.611009164	0.512081581	0.007086584	37.00337646
GCST90017028	Genus Lachnospiraceae UCG010	Digestive system disorders of fetus and newborn	Weighted median	10	1.083432233	0.957899568	.258034079	−0.79405092	2.960915387	2.954803743	0.452010025	19.31564495
GCST90017028	Genus Lachnospiraceae UCG010	Digestive system disorders of fetus and newborn	Simple mode	10	0.8	NA	.021484375	NA	NA	2.225540928	NA	NA
GCST90017028	Genus Lachnospiraceae UCG010	Digestive system disorders of fetus and newborn	Weighted mode	10	0.360462626	3.016035083	.904867044	−5.550966136	6.271891388	1.433992663	0.003883703	529.4778794
GCST90017042	Genus *Peptococcus*	Digestive system disorders of fetus and newborn	Inverse variance weighted	12	0.769040093	0.389347343	.0482451	0.005919301	1.532160884	2.157694074	1.005936855	4.628166959
GCST90017042	Genus *Peptococcus*	Digestive system disorders of fetus and newborn	MR Egger	12	−0.555652722	1.495467898	.717973723	−3.486769802	2.375464358	0.573697674	0.030599555	10.75600667
GCST90017042	Genus *Peptococcus*	Digestive system disorders of fetus and newborn	Weighted median	12	0.575381124	0.51013935	.259366335	−0.424492003	1.57525425	1.777807962	0.654101982	4.831969992
GCST90017042	Genus *Peptococcus*	Digestive system disorders of fetus and newborn	Simple mode	12	0.666666667	NA	.038574219	NA	NA	1.947734041	NA	NA
GCST90017042	Genus *Peptococcus*	Digestive system disorders of fetus and newborn	Weighted mode	12	0.620430142	1.996488149	.755982515	−3.29268663	4.533546913	1.859727816	0.037153896	93.08815182
GCST90017075	Genus Tyzzerella3	Digestive system disorders of fetus and newborn	Inverse variance weighted	13	0.856300797	0.399324443	.032002963	0.073624888	1.638976705	2.354435031	1.076402958	5.149896953
GCST90017075	Genus Tyzzerella3	Digestive system disorders of fetus and newborn	MR Egger	13	0.301715004	2.38527986	.901626213	−4.373433523	4.97686353	1.352175806	0.012607877	145.0188204
GCST90017075	Genus Tyzzerella3	Digestive system disorders of fetus and newborn	Weighted median	13	0.906267556	0.535552206	.090605671	−0.143414769	1.955949881	2.475067222	0.866394641	7.070632091
GCST90017075	Genus Tyzzerella3	Digestive system disorders of fetus and newborn	Simple mode	13	0.384615385	NA	.266845703	NA	NA	1.469049194	NA	NA
GCST90017075	Genus Tyzzerella3	Digestive system disorders of fetus and newborn	Weighted mode	13	0.736737962	1.80536087	.683212382	−2.801769343	4.275245266	2.089109632	0.060702564	71.89777125
GCST90017116	Phylum Proteobacteria	Digestive system disorders of fetus and newborn	Simple mode	12	−1	NA	.000488281	NA	NA	0.367879441	NA	NA
GCST90017116	Phylum Proteobacteria	Digestive system disorders of fetus and newborn	Inverse variance weighted	12	−1.468623981	0.68418803	.031831434	−2.80963252	−0.127615442	0.230242085	0.060227121	0.880191799
GCST90017116	Phylum Proteobacteria	Digestive system disorders of fetus and newborn	MR Egger	12	−0.613484427	1.896584012	.753003756	−4.330789092	3.103820237	0.541460897	0.013157161	22.28291491
GCST90017116	Phylum Proteobacteria	Digestive system disorders of fetus and newborn	Weighted median	12	−1.391794606	0.875497767	.111898372	−3.10777023	0.324181017	0.248628713	0.044700516	1.382897613
GCST90017116	Phylum Proteobacteria	Digestive system disorders of fetus and newborn	Weighted mode	12	−1.424398573	3.653327755	.69661736	−8.584920973	5.736123827	0.240653155	0.000186903	309.8610054

If the *P*-value is <.05, it is generally considered statistically significant.

lo_ci = lower 95% confidence interval; MR = Mendelian Randomization; OR = odds ratio; OR_lci95 = lower 95% confidence interval for odds ratio; OR_uci95 = upper 95% confidence interval for odds ratio; up_ci = upper 95% confidence interval.

**Figure 3.**
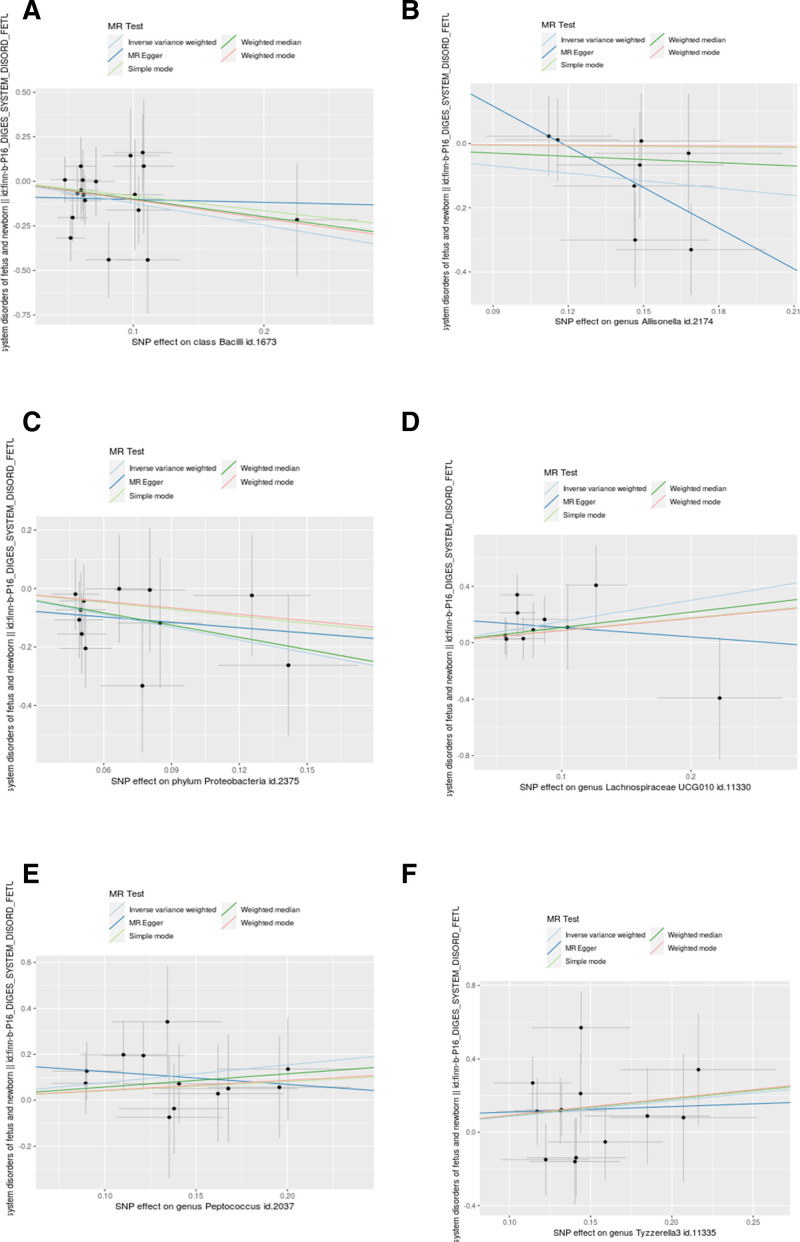
A suite of 5 different MR methodologies was applied to scrutinize the potential causal relationship between gut microbial communities and neonatal DSD. (A–F) Casual association between class Bacilli, genus *Allisonella*, genus Lachnospiraceae UCG010, genus *Peptococcus*, genus Tyzzerella3, phylum Proteobacteria, and neonatal DSD. MR = Mendelian randomization, SNPs = single nucleotide polymorphisms.

**Figure 4.**
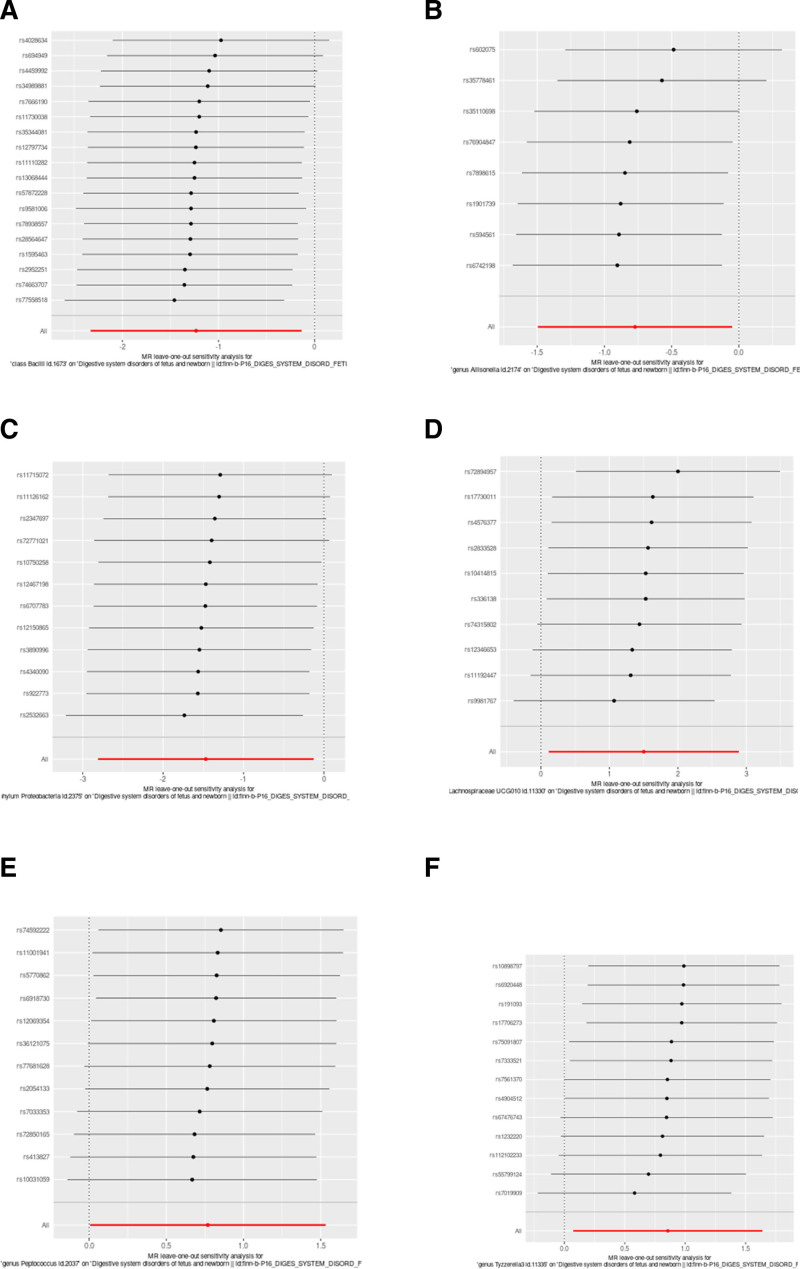
Results of the leave-one-out sensitivity analysis of MR result between the gut microbiota and DSD. (A–F) Leave-one-out anslysis among class Bacilli, genus *Allisonella*, genus Lachnospiraceae UCG010, genus *Peptococcus*, genus Tyzzerella3, phylum Proteobacteria, and DSD. MR = Mendelian randomization.

**Figure 5.**
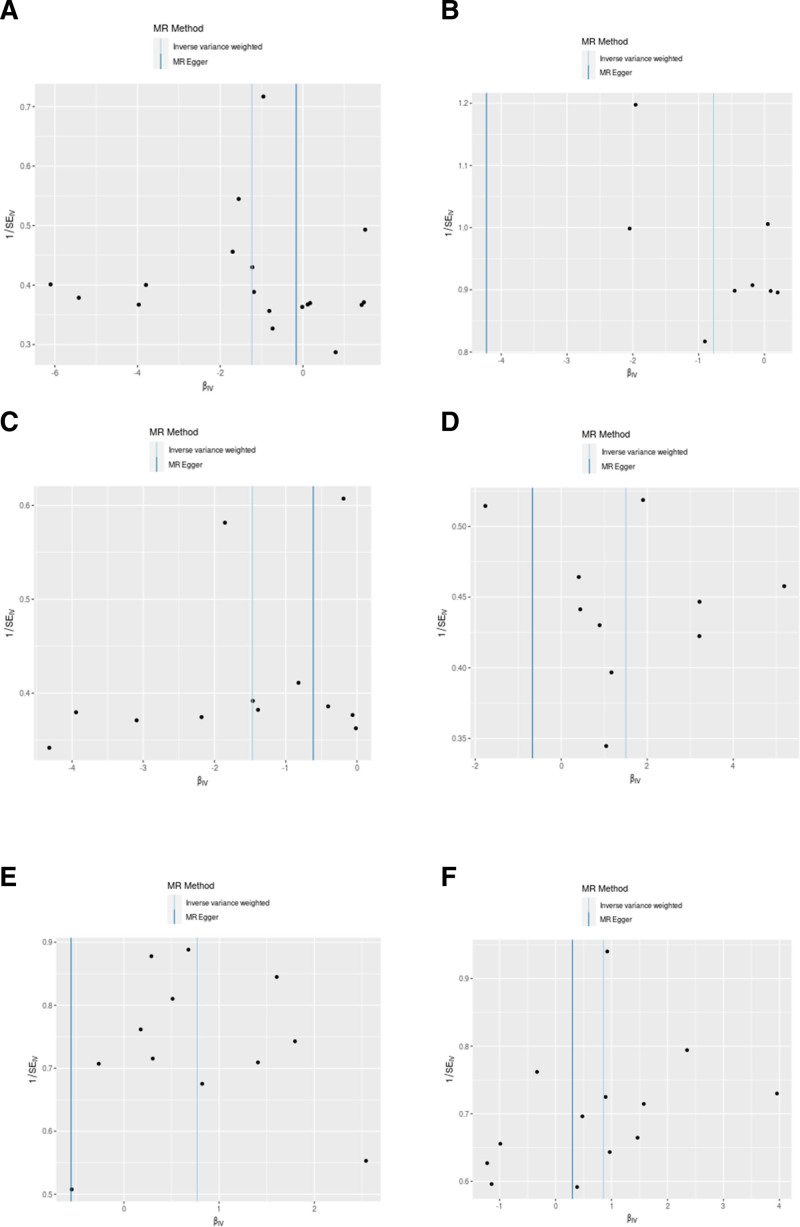
Funnel plot test of MR results between the gut microbiota and DSD. (A–F) Funnel plot test among class Bacilli, genus *Allisonella*, genus Lachnospiraceae UCG010, genus *Peptococcus*, genus Tyzzerella3, phylum Proteobacteria, and DSD. MR = Mendelian randomization.

**Figure 6.**
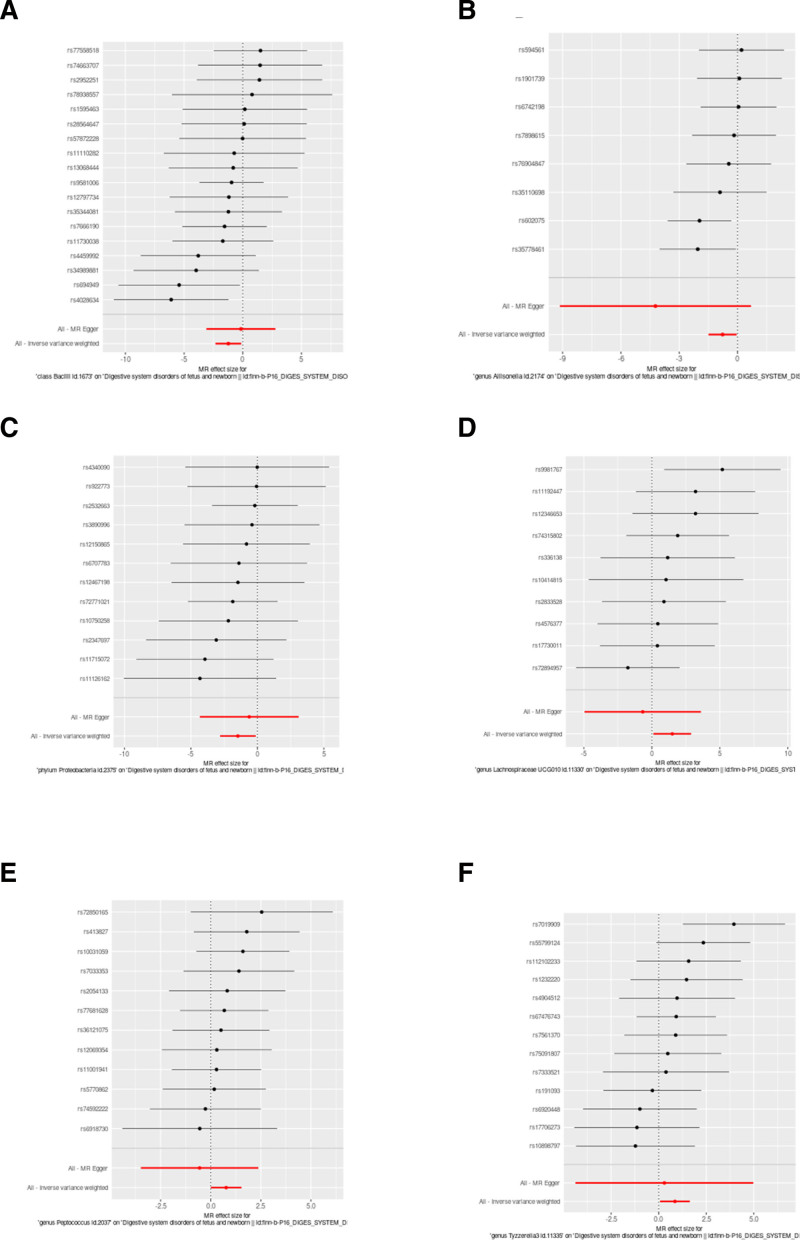
The potential roles of different SNPs in the causal relationship between the gut microbiota and DSD. (A–F) Random forest plot of SNPs of IVW analysis among class Bacilli, genus *Allisonella*, genus Lachnospiraceae UCG010, genus *Peptococcus*, genus Tyzzerella3, phylum Proteobacteria, and DSD. IVW = inverse variance weighted, MR = Mendelian randomization, SNPs = single nucleotide polymorphisms.

The heterogeneity checks, horizontal pleiotropy evaluations, and leave-one-out analyses for these 6 microbial groups showed strong reliability, the *P*-values, all above .05, suggest that there is no significant evidence of heterogeneity or pleiotropy (Table 2 and Figs. 3–6). Moreover, the Steiger directionality test (Table 2) revealed no evidence of reverse causation, thereby reinforcing the forward causal relationships identified in our analysis. These findings support a directional causal relationship between specific gut microbiota and the risk of neonatal DSD.

**Table 2:** Sensitivity analysis of gut microbiome and digestive system disorders of fetus and newborn.

Exposure	Heterogeneity tests				Test for directional horizontal pleiotropy			Test that the exposure is upstream of the outcome			
	Method	Q	Q_df	Q_pval	egger_intercept	se	pval	snp_r2.exposure	snp_r2.outcome	correct_causal_direction	steiger_pval
Class Bacilli											
Digestive system disorders of fetus and newborn	MR Egger	12.79133	16	0.6879494	−0.0856013	0.1112835	0.4529701	5.01E−05	0	TRUE	0.397302
	Inverse variance weighted	13.38303	17	0.7101911							
Genus *Allisonella*											
Digestive system disorders of fetus and newborn	MR Egger	4.136743	6	0.6581774	0.4966225	0.3568969	0.213473	2.23E−05	0	TRUE	0.5721202
	Inverse variance weighted	6.073018	7	0.5312497							
Genus Lachnospiraceae											
Digestive system disorders of fetus and newborn	MR Egger	6.296801	8	0.6140237	0.1751516	0.1666487	0.3239571	2.75E−05	0	TRUE	0.5305075
	Inverse variance weighted	7.40145	9	0.5953983							
Genus *Peptococcus*											
Digestive system disorders of fetus and newborn	MR Egger	2.932933	10	0.9829586	0.1799633	0.1961573	0.3805048	4.22E−05	0	TRUE	0.4369878
	Inverse variance weighted	3.774636	11	0.9760517							
Genus Tyzzerella3											
Digestive system disorders of fetus and newborn	MR Egger	12.43525	11	0.3318313	0.079424	0.3363673	0.8176764	3.68E−05	0	TRUE	0.4683044
	Inverse variance weighted	12.49828	12	0.4065361							
Phylum Proteobacteria											
Digestive system disorders of fetus and newborn	MR Egger	3.487305	10	0.9675277	−0.0605385	0.1252251	0.6391959	3.01E−05	0	TRUE	0.5115248
	Inverse variance weighted	3.721017	11	0.977379							

Q_pval >0.05 indicates no heterogeneity. Egger_intercept pval >0.05 indicates no horizontal pleiotropy. Steiger_pval >0.05 indicates no causal relationship in Mendelian randomization analysis.

## 4. Discussion

The present study has discovered 6 specific gut microbiota taxa that are causally related to neonatal DSD. Genus Lachnospiraceae UCG010, genus *Peptococcus*, and genus Tyzzerella3 showed positive causal relationships, this suggests that a higher relative abundance is linked to an elevated risk for neonatal DSD. In contrast, class Bacilli, genus *Allisonella*, and phylum Proteobacteria exhibited negative causal relationships, with increased abundance linked to a reduced risk of neonatal DSD. Sensitivity analyses confirmed the robustness of these causal associations.

MR uses genetic data to assess causal relationships, akin to a “natural” randomized controlled trial. This method is less susceptible to traditional confounding factors and ensures a proper temporal sequence of causality,^[33,34]^ thus providing a more reliable assessment of the relationship between the gut microbiome and neonatal DSD. Our MR analysis corroborates findings from traditional studies, which also indicate gut microbiota dysbiosis in neonatal DSD patients or animal models.^[35]^ However, the specific dysbiotic taxa identified in previous studies have varied, highlighting ongoing debate in the field. Our study uniquely identifies class Bacilli, genus *Allisonella*, phylum Proteobacteria, genus Lachnospiraceae UCG010, genus *Peptococcus*, and genus Tyzzerella3 as playing roles in neonatal DSD, providing new insights into its pathogenesis.

### 4.1. Specific microbiota insights

Class Bacilli, including beneficial *Lactobacillus* and *Bifidobacterium* species, are crucial for neonatal gut health. *Lactobacillus* aids in lactose digestion, energy provision, and harmful bacteria prevention, while also modulating the immune system by promoting cell division, antibody production, macrophage activation, and interferon production.^[36]^ *Bifidobacterium*, a high-abundance Gram-positive bacterium in the neonatal gut, aids in digestion, immune enhancement, and pathogen inhibition, and produces aromatic lactic acids impacting early immune function.^[37]^ Bifidobacteria convert aromatic amino acids into aromatic lactic acids through an unidentified enzyme, aromatic lactic dehydrogenase. The resulting indole lactic acid can activate the aryl hydrocarbon receptor in vitro.^[38]^ The aryl hydrocarbon receptor is instrumental in sustaining intestinal balance in neonates and controlling immune system responses.^[39]^ These findings underscore the beneficial roles of these bacteria in neonatal digestive health.

Phylum Proteobacteria, including various commensal bacteria, also plays a role in immune regulation. In early neonatal colonization, facultative anaerobes like Proteobacteria prepare the gut environment for strict anaerobes, contributing to a stable and low-reactivity microbiome.^[40–44]^ This transition is essential for healthy gut development.^[45,46]^

Genus *Allisonella* role in digestive diseases needs further investigation, despite its potential involvement suggested by some studies.^[11]^ Genus Tyzzerella3 has been linked to Crohn disease,^[47]^ indicating its possible involvement in DSD. Understanding its role in neonatal gastrointestinal disorders could be crucial for preventive measures.

### 4.2. Potential mechanisms and further research

Gut microbiota influences various processes, including bile acid composition, aromatic hydrocarbon metabolism, and short-chain fatty acid production.^[35,48]^ For example, *Peptococcus* has been implicated in gallstone disease susceptibility by mediating omega-3 polyunsaturated fatty acids concentration reduction.^[49]^ Lachnospiraceae, this key element of the gut microbiome is correlated with obesity, attributed to its generation of short-chain fatty acids, particularly butyrate, essential for microbial and epithelial cell growth.^[50–52]^ These microbiota changes are linked to neonatal gastrointestinal disease risks, such as glycogen storage disease and obesity,^[53]^ warranting further research into their specific roles and mechanisms.

This study has several limitations: Our findings were derived from datasets of European ancestry, thus caution is necessary when generalizing these associations to non-European populations. Future studies using diverse population datasets are essential to confirm the generalizability of these results. The relationship between gut microbiota and neonatal DSD is multifactorial, necessitating consideration of environmental and behavioral factors. Larger MR studies or randomized controlled trials are needed to obtain more accurate results. Our study used datasets with significantly imbalanced case-control proportions (143 neonatal DSD cases vs 218,649 controls). Such imbalances can introduce potential biases, including overfitting or artificially inflated statistical significance. Future analyses should aim to utilize datasets with more balanced case-control proportions or apply statistical adjustments to mitigate this potential bias. Our analysis did not apply multiple-testing adjustment, which may elevate the chance of Type I errors. As this work is exploratory and aims to generate hypotheses for future study, findings should be interpreted with caution.

## 5. Conclusions

The neonatal gut microbiome composition and its shifts are significantly impactful on health outcomes, with different microbiota taxa playing critical roles in the development of DSD. Further research into these microbiota and their metabolites is essential for understanding, preventing, and treating neonatal gastrointestinal diseases.

## Author contributions

**Conceptualization:** Jian Pang, Shuihua Yang.

**Data curation:** Jian Pang.

**Methodology:** Zuojian Yang, Pingping Liu.

**Software:** Zuojian Yang, Pingping Liu, Guidan He.

**Supervision:** Shuihua Yang.

**Visualization:** Baoxing Pan, Guidan He.

**Writing – original draft:** Jian Pang.

**Writing – review & editing:** Jian Pang, Shuihua Yang.

## Supplementary Material


