# Supplementary figures and images for: Causal association between gut microbiota and neonatal digestive system diseases: A Mendelian randomization study
Source: Medicine (Baltimore). 2025 Aug 15;104(33):e44009. doi: 10.1097/MD.0000000000044009 (PMC12367028; doi:10.1097/MD.0000000000044009)

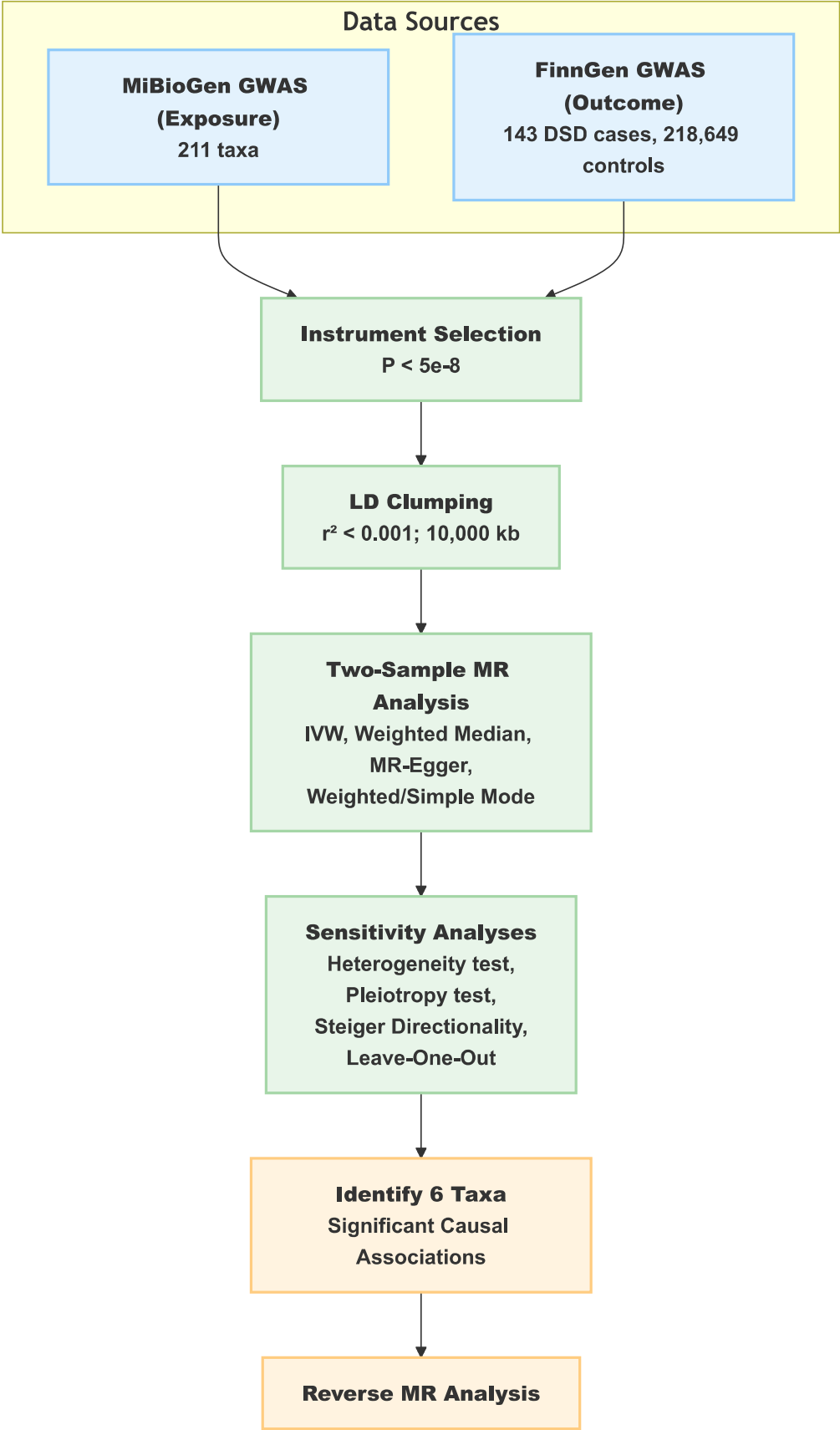

Supplement: Supplementary file 1 [file medi-104-e44009-s001.pdf]
